# 2-[*N*-(4-{4-[(2-Hy­droxy-5-meth­oxy­benzyl­idene)amino]­benz­yl}phen­yl)carboximido­yl]-4-meth­oxy­phenol

**DOI:** 10.1107/S1600536812010550

**Published:** 2012-03-24

**Authors:** Ali Ourari, Lotfi Baameur, Gilles Bouet, Magali Allain

**Affiliations:** a Laboratoire d’Electrochimie, d’Ingénierie Moléculaire et de Catalyse Redox (LEIMCR), Faculté des Sciences de l’Ingénieur, Université Farhat Abbas, Sétif 19000, Algeria; b Laboratoire SONAS, E.A. 921, Faculté de Pharmacie, 16 Boulevard Daviers, 49045 Angers Cedex 01, France; c MOLTECH ANJOU UMR CNRS 6200, 2, bd Lavoisier, 49045 Angers Cedex, France

## Abstract

In the title Schiff base, C_29_H_26_N_2_O_4_, the complete molecule is generated by a crystallographic twofold axis and is V-shaped. The planes of the benzene rings of the central diphenyl­methane unit make a dihedral angle of 78.11 (4)° while adjacent benzene and 5-meth­oxy­salicyl­idene rings are twisted with respect to each other by a dihedral angle of 11.84 (8)°. The Schiff base is in the enol–imino form and an intra­molecular O—H⋯N hydrogen bond is observed.

## Related literature

For related bis-bidentate Schiff base ligand structures, see: Birkedal & Pattison (2006 ▶); Shahverdizadeh & Tiekink (2011 ▶). For Schiff base ligands, see: Chu & Huang (2007 ▶); Yoshida & Ichikawa, (1997 ▶); Kruger *et al.* (2001 ▶); Moutet & Ourari (1997 ▶). For applications of bis-bidentate Schiff base ligands, see: Lin *et al.* (2008 ▶); Sadeghi *et al.* (2003 ▶).
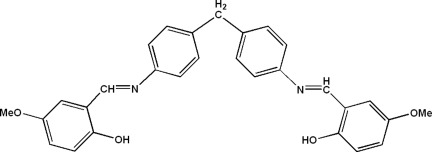


## Experimental

### 

#### Crystal data

C_29_H_26_N_2_O_4_
*M* *_r_* = 466.52
Monoclinic, 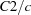

*a* = 41.307 (4) Å
*b* = 4.5993 (3) Å
*c* = 12.2229 (13) Å
β = 93.653 (12)°
*V* = 2317.4 (4) Å^3^
*Z* = 4
Mo *K*α radiation
μ = 0.09 mm^−1^
*T* = 293 K
0.69 × 0.38 × 0.06 mm

#### Data collection

Stoe IPDS diffractometer
Absorption correction: gaussian (*ABSGAUSS* in *PLATON*; Spek, 2009 ▶) *T*_min_ = 0.953, *T*_max_ = 0.993
10694 measured reflections
2244 independent reflections
1662 reflections with *I* > 2σ(*I*)
*R*_int_ = 0.037

#### Refinement

*R*[*F* ^2^ > 2σ(*F* ^2^)] = 0.039
*wR*(*F* ^2^) = 0.116
*S* = 1.06
2244 reflections
160 parameters
H-atom parameters constrained
Δρ_max_ = 0.17 e Å^−3^
Δρ_min_ = −0.15 e Å^−3^

### 

Data collection: *EXPOSE* (Stoe & Cie, 1995 ▶); cell refinement: *X-RED* (Stoe & Cie, 1995 ▶); data reduction: *X-RED*; program(s) used to solve structure: *SIR92* (Altomare *et al.*, 1993 ▶); program(s) used to refine structure: *SHELXL97* (Sheldrick, 2008 ▶); molecular graphics: *ORTEP-3 for Windows* (Farrugia, 1997 ▶); software used to prepare material for publication: *publCIF* (Westrip, 2010 ▶).

## Supplementary Material

Crystal structure: contains datablock(s) global, I. DOI: 10.1107/S1600536812010550/qk2024sup1.cif

Structure factors: contains datablock(s) I. DOI: 10.1107/S1600536812010550/qk2024Isup2.hkl

Additional supplementary materials:  crystallographic information; 3D view; checkCIF report

## Figures and Tables

**Table 1:** Hydrogen-bond geometry (Å, °)

*D*—H⋯*A*	*D*—H	H⋯*A*	*D*⋯*A*	*D*—H⋯*A*
O1—H1⋯N1	0.89	1.81	2.6177 (15)	151

**Fig. 1.**
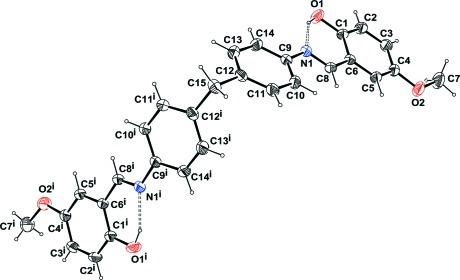
A molecule of the title compound with the atom-labelling scheme. Displacement ellipsoids are drawn at the 50% probability level. H atoms are represented as small spheres of arbitrary radii. Hydrogen bonds are shown as dashed lines. [Symmetry code: (i) -x + 1, y, -z + 3/2]

## supplementary crystallographic information

### Comment

Bis-bidentate Schiff base ligands have been extensively studied and used as building blocks in metallo-supramolecular chemistry (Birkedal & Pattison, 2006; Shahverdizadeh & Tiekink, 2011; Chu & Huang, 2007; Yoshida & Ichikawa, 1997; Kruger *et al.*, 2001). These compounds were also used as thermosetting resins (Lin *et al.*, 2008) and in ion selective membranes for detecting traces of copper (Sadeghi *et al.*, 2003). We were interested in such ligands owing to their diverse applications in coordination chemistry, catalysis and electrocatalysis (Moutet & Ourari, 1997).

The molecule of the title compound is arranged around the two fold axis at 1/2, *y*, 3/4 of the unit cell and methylene carbon C14 coinciding with it. The molecule is V-shaped and has a dihedral angle of 78.11 (4)° between the two inner phenyl rings. The phenyl and the 5-methoxysalicylidene rings are slightly twisted with respect to each other by a dihedral angle of 11.84 (8)°. There are two symmetry equivalent intramolecular O-H···N hydrogen bonds. The bond lengths and bond angles within the molecule agree well with those of the closely related compounds C_27_H_22_N_2_O_2_ (CCDC refcode YEFWUC; Birkedal & Pattison, 2006) and C_26_H_20_N_2_O_3_ (Shahverdizadeh & Tiekink, 2011). In the unit cell, the molecules are tightly stacked one above the other along the short *b-*axis (*b* = 4.5993 (3) Å) and are held together in this direction by slipped π-π stacking interactions between the phenyl rings and the iminomethylidene groups. The architecture and space group of the title structure is identical with CCDC YEFWUC.

### Experimental

5-Methoxysalicyaldehyde (98%), 4, 4'-diaminodiphenylmethane (97%), anhydrous ethanol were all purchased from Alfa aesar and used as received. 200 mg (1 mmol) of 4, 4'-diaminodiphenylmethane were dissolved in 10 ml of absolute ethanol. To this solution, 304 mg (2 mmol) of 5-methoxysalicyaldehyde in 5 ml of absolute ethanol was dropwisely added under stirring. Then, this mixture was heated for 15 min at 50 °C. The resulting yellow precipitate was recovered by filtration, washed several times with a small portions of EtOH and then with diethyl ether to give 443 mg (95%) of the title compound. Suitable crystals were obtained by slow evaporation of a solution in dichloromethane/ethanol (9/1, v /v).

### Refinement

All H atoms attached to C were fixed geometrically and treated as riding with C—H = 0.96 Å (methyl), 0.92 Å (methylene) or 0.93 Å (aromatic) with *U*_iso_(H) = 1.2*U*_eq_(C) or *U*_iso_(H) = 1.5*U*_eq_(methyl). The H atom of the hydroxyl group was initially refined using a soft restraint O—H = 0.89 (1) Å and *U*_iso_(H) = 1.2*U*_eq_(O). Then, in the last cycles of refinement, it was treated as riding on its parent O atom.

### Crystal data

**Table:**

C_29_H_26_N_2_O_4_	*F*(000) = 984
*M**_r_* = 466.52	*D*_x_ = 1.337 Mg m^−^^3^
Monoclinic, *C*2/*c*	Mo *K*α radiation, λ = 0.71073 Å
Hall symbol: -C 2yc	Cell parameters from 8000 reflections
*a* = 41.307 (4) Å	θ = 2.0–25.9°
*b* = 4.5993 (3) Å	µ = 0.09 mm^−^^1^
*c* = 12.2229 (13) Å	*T* = 293 K
β = 93.653 (12)°	Plate, yellow
*V* = 2317.4 (4) Å^3^	0.69 × 0.38 × 0.06 mm
*Z* = 4	

### Data collection

**Table:**

Stoe IPDS diffractometer	2244 independent reflections
Radiation source: normal-focus sealed tube	1662 reflections with *I* > 2σ(*I*)
Graphite monochromator	*R*_int_ = 0.037
Detector resolution: 6.66 pixels mm^-1^	θ_max_ = 25.8°, θ_min_ = 2.0°
0.6° φ scans	*h* = −50→50
Absorption correction: gaussian (*PLATON*-ABSGAUSS; Spek, 2009)	*k* = −5→5
*T*_min_ = 0.953, *T*_max_ = 0.993	*l* = −14→15
10694 measured reflections	

### Refinement

**Table:**

Refinement on *F*^2^	Primary atom site location: structure-invariant direct methods
Least-squares matrix: full	Secondary atom site location: difference Fourier map
*R*[*F*^2^ > 2σ(*F*^2^)] = 0.039	Hydrogen site location: inferred from neighbouring sites
*wR*(*F*^2^) = 0.116	H-atom parameters constrained
*S* = 1.06	*w* = 1/[σ^2^(*F*_o_^2^) + (0.072*P*)^2^ + 0.1658*P*] where *P* = (*F*_o_^2^ + 2*F*_c_^2^)/3
2244 reflections	(Δ/σ)_max_ = 0.001
160 parameters	Δρ_max_ = 0.17 e Å^−^^3^
0 restraints	Δρ_min_ = −0.15 e Å^−^^3^

### Special details

**Table:**

Geometry. All e.s.d.'s (except the e.s.d. in the dihedral angle between two l.s. planes) are estimated using the full covariance matrix. The cell e.s.d.'s are taken into account individually in the estimation of e.s.d.'s in distances, angles and torsion angles; correlations between e.s.d.'s in cell parameters are only used when they are defined by crystal symmetry. An approximate (isotropic) treatment of cell e.s.d.'s is used for estimating e.s.d.'s involving l.s. planes.
Refinement. Refinement of *F*^2^ against ALL reflections. The weighted *R*-factor *wR* and goodness of fit *S* are based on *F*^2^, conventional *R*-factors *R* are based on *F*, with *F* set to zero for negative *F*^2^. The threshold expression of *F*^2^ > σ(*F*^2^) is used only for calculating *R*-factors(gt) *etc*. and is not relevant to the choice of reflections for refinement. *R*-factors based on *F*^2^ are statistically about twice as large as those based on *F*, and *R*- factors based on ALL data will be even larger.

### Fractional atomic coordinates and isotropic or equivalent isotropic displacement parameters (Å^2^)

**Table:**

	*x*	*y*	*z*	*U*_iso_*/*U*_eq_	
O1	0.37635 (3)	0.9988 (3)	0.40329 (8)	0.0634 (3)	
H1	0.3881	0.8694	0.4423	0.076*	
O2	0.28937 (3)	1.4522 (3)	0.68223 (9)	0.0651 (4)	
N1	0.40032 (2)	0.7019 (2)	0.57231 (8)	0.0404 (3)	
C1	0.35488 (3)	1.1095 (3)	0.47152 (10)	0.0435 (3)	
C2	0.33158 (3)	1.3024 (4)	0.43070 (11)	0.0521 (4)	
H2	0.3309	1.3538	0.3570	0.063*	
C3	0.30928 (3)	1.4206 (3)	0.49763 (12)	0.0502 (4)	
H3	0.2936	1.5494	0.4687	0.060*	
C4	0.31021 (3)	1.3469 (3)	0.60828 (11)	0.0451 (3)	
C5	0.33363 (3)	1.1561 (3)	0.64977 (11)	0.0438 (3)	
H5	0.3345	1.1092	0.7239	0.053*	
C6	0.35595 (3)	1.0324 (3)	0.58312 (10)	0.0377 (3)	
C7	0.26320 (4)	1.6273 (4)	0.64047 (16)	0.0725 (5)	
H7A	0.2715	1.8012	0.6091	0.109*	
H7B	0.2498	1.6779	0.6989	0.109*	
H7C	0.2506	1.5214	0.5851	0.109*	
C8	0.37983 (3)	0.8291 (3)	0.63036 (10)	0.0405 (3)	
H8	0.3803	0.7902	0.7051	0.049*	
C9	0.42351 (3)	0.5050 (3)	0.62024 (10)	0.0381 (3)	
C10	0.42235 (3)	0.3824 (3)	0.72409 (11)	0.0460 (3)	
H10	0.4053	0.4280	0.7673	0.055*	
C11	0.44638 (3)	0.1933 (3)	0.76319 (11)	0.0463 (3)	
H11	0.4450	0.1111	0.8323	0.056*	
C12	0.47241 (3)	0.1226 (3)	0.70280 (11)	0.0411 (3)	
C13	0.47310 (3)	0.2425 (3)	0.59852 (12)	0.0489 (4)	
H13	0.4902	0.1979	0.5556	0.059*	
C14	0.44889 (3)	0.4265 (3)	0.55754 (11)	0.0467 (4)	
H14	0.4496	0.4989	0.4867	0.056*	
C15	0.5000	−0.0659 (4)	0.7500	0.0474 (5)	
H15A	0.4922	−0.1658	0.8076	0.057*	

### Atomic displacement parameters (Å^2^)

**Table:**

	*U*^11^	*U*^22^	*U*^33^	*U*^12^	*U*^13^	*U*^23^
O1	0.0703 (7)	0.0835 (8)	0.0375 (5)	0.0297 (6)	0.0109 (5)	0.0061 (5)
O2	0.0592 (6)	0.0793 (8)	0.0583 (6)	0.0268 (6)	0.0149 (5)	0.0052 (6)
N1	0.0401 (5)	0.0405 (6)	0.0402 (6)	0.0025 (5)	−0.0008 (4)	−0.0006 (5)
C1	0.0460 (7)	0.0490 (8)	0.0353 (6)	0.0034 (6)	0.0015 (5)	−0.0016 (6)
C2	0.0586 (8)	0.0599 (10)	0.0372 (7)	0.0101 (7)	−0.0021 (6)	0.0062 (6)
C3	0.0472 (7)	0.0528 (9)	0.0496 (8)	0.0094 (6)	−0.0055 (6)	0.0050 (6)
C4	0.0417 (6)	0.0468 (8)	0.0471 (7)	0.0038 (6)	0.0050 (6)	−0.0013 (6)
C5	0.0463 (7)	0.0481 (8)	0.0370 (6)	0.0028 (6)	0.0029 (5)	0.0038 (6)
C6	0.0387 (6)	0.0378 (7)	0.0363 (6)	−0.0012 (5)	−0.0011 (5)	−0.0008 (5)
C7	0.0619 (10)	0.0752 (12)	0.0819 (12)	0.0267 (9)	0.0173 (9)	0.0054 (10)
C8	0.0444 (7)	0.0419 (8)	0.0348 (6)	0.0008 (6)	−0.0003 (5)	0.0013 (5)
C9	0.0380 (6)	0.0358 (7)	0.0399 (6)	−0.0009 (5)	−0.0026 (5)	−0.0017 (5)
C10	0.0411 (7)	0.0519 (9)	0.0451 (7)	0.0042 (6)	0.0050 (5)	0.0047 (6)
C11	0.0462 (7)	0.0477 (8)	0.0444 (7)	−0.0017 (6)	−0.0020 (6)	0.0080 (6)
C12	0.0393 (7)	0.0312 (7)	0.0516 (7)	−0.0039 (5)	−0.0059 (5)	−0.0040 (6)
C13	0.0471 (7)	0.0487 (9)	0.0515 (8)	0.0076 (6)	0.0075 (6)	−0.0037 (6)
C14	0.0518 (7)	0.0485 (9)	0.0401 (7)	0.0070 (6)	0.0055 (6)	0.0015 (6)
C15	0.0445 (10)	0.0342 (11)	0.0626 (12)	0.000	−0.0055 (9)	0.000

### Geometric parameters (Å, º)

**Table:**

O1—C1	1.3548 (15)	C7—H7B	0.9600
O1—H1	0.8879	C7—H7C	0.9600
O2—C4	1.3754 (16)	C8—H8	0.9300
O2—C7	1.4168 (19)	C9—C14	1.3852 (17)
N1—C8	1.2802 (16)	C9—C10	1.3926 (18)
N1—C9	1.4176 (16)	C10—C11	1.3822 (19)
C1—C2	1.379 (2)	C10—H10	0.9300
C1—C6	1.4074 (18)	C11—C12	1.3814 (18)
C2—C3	1.3819 (19)	C11—H11	0.9300
C2—H2	0.9300	C12—C13	1.3908 (19)
C3—C4	1.392 (2)	C12—C15	1.5161 (17)
C3—H3	0.9300	C13—C14	1.3795 (19)
C4—C5	1.3787 (19)	C13—H13	0.9300
C5—C6	1.3901 (18)	C14—H14	0.9300
C5—H5	0.9300	C15—C12^i^	1.5161 (17)
C6—C8	1.4520 (18)	C15—H15A	0.9160
C7—H7A	0.9600		
			
C1—O1—H1	106.2	H7B—C7—H7C	109.5
C4—O2—C7	117.30 (12)	N1—C8—C6	122.03 (12)
C8—N1—C9	121.08 (11)	N1—C8—H8	119.0
O1—C1—C2	119.22 (12)	C6—C8—H8	119.0
O1—C1—C6	121.38 (12)	C14—C9—C10	118.04 (12)
C2—C1—C6	119.40 (12)	C14—C9—N1	116.95 (11)
C1—C2—C3	121.01 (13)	C10—C9—N1	125.00 (11)
C1—C2—H2	119.5	C11—C10—C9	120.28 (12)
C3—C2—H2	119.5	C11—C10—H10	119.9
C2—C3—C4	120.06 (13)	C9—C10—H10	119.9
C2—C3—H3	120.0	C12—C11—C10	122.00 (13)
C4—C3—H3	120.0	C12—C11—H11	119.0
O2—C4—C5	115.87 (12)	C10—C11—H11	119.0
O2—C4—C3	124.94 (13)	C11—C12—C13	117.28 (12)
C5—C4—C3	119.19 (12)	C11—C12—C15	121.54 (11)
C4—C5—C6	121.39 (12)	C13—C12—C15	121.11 (11)
C4—C5—H5	119.3	C14—C13—C12	121.29 (12)
C6—C5—H5	119.3	C14—C13—H13	119.4
C5—C6—C1	118.95 (12)	C12—C13—H13	119.4
C5—C6—C8	119.31 (11)	C13—C14—C9	121.04 (13)
C1—C6—C8	121.75 (11)	C13—C14—H14	119.5
O2—C7—H7A	109.5	C9—C14—H14	119.5
O2—C7—H7B	109.5	C12^i^—C15—C12	110.27 (15)
H7A—C7—H7B	109.5	C12^i^—C15—H15A	106.7
O2—C7—H7C	109.5	C12—C15—H15A	106.6
H7A—C7—H7C	109.5		
			
C6—C8—N1—C9	179.75 (11)		

Symmetry code: (i) −*x*+1, *y*, −*z*+3/2.

### Hydrogen-bond geometry (Å, º)

**Table:**

*D*—H···*A*	*D*—H	H···*A*	*D*···*A*	*D*—H···*A*
O1—H1···N1	0.89	1.81	2.6177 (15)	151
